# O036. Cocaine and headache: a 2-year follow-up study in chronic cocaine users and literature review

**DOI:** 10.1186/1129-2377-16-S1-A167

**Published:** 2015-09-28

**Authors:** Luisa Fofi, Valerio Orlandi, Nicola Vanacore, Maria C Mizzoni, Alba Rosa, Cinzia Aurilia, Gabriella Egeo, Pietro Casella, Piero Barbanti

**Affiliations:** Headache and Pain Unit, Department of Neurological, Motor and Sensorial Sciences, IRCCS San Raffaele Pisana, Rome, Italy; Drug Addiction Service, 20th District, UOS Municipio 17, ASL RME, Rome, Italy; National Centre of Epidemiology, Surveillance and Health Promotion, National Institute of Health, Rome, Italy

As many as 14-21 million people worldwide (0.3-0.5% of the population, aged 15-65 years) use cocaine [1]. In Europe, cocaine consumption has shown a 2- to 3-fold increase during the last 2 decades [2, 3]; in Italy, lifetime cocaine experience among adults corresponds to 6.6% [4]. Cocaine use and headache share some common characteristics: present heavy global burden, prevail among young individuals, cause more severe consequences in females, may lead to emergency department access and progress to chronification. The study of headache in chronic cocaine users (CCU) is of interest also from a pathophysiological point of view, given that chronic cocaine use causes decreased dopamine and serotonin synaptic levels, a typical migraine biochemical feature (“empty neuron” condition) [5, 6]. In a previous study we encouraged clinicians to carry out a more in-depth investigation on cocaine use in all headache suffers, especially those with migraine, as headache occurs in a very high proportion of CCU (90%), mostly showing migraine or migraine-like characteristics, while cocaine-induced headache, as classified by the ICHD criteria [7], seems exceedingly rare (2.2%). Moreover, we pointed out that CCU sometimes use cocaine as an acute remedy for the headache attack, even though improvement occurs very rarely (17.2% of cases) [8]. Recently, it has been described that patients with intractable cluster headache who tried cocaine, being dissatisfied with conventional treatments in terms of efficacy and/or tolerability, referred a full or partial improvement in 30.8% of cases [9]. The present study was aimed to evaluate the modification of the clinical characteristics of headache in CCU after a 2-year follow-up period. We contacted by phone the 80 patients previously enrolled [8] attending the Cocaine Addiction Service of the Drug Addiction Service, 20th District, Rome. Of these 80 patients, 60 (still followed by the Drug Addiction Service) were enrolled and interviewed by the same physicians of the previous study. We studied the modifications of headache pattern and characteristics relative to their actual cocaine consumption in CCU patients previously subdivided into 3 groups: neither lifetime nor current headache (group 0); lifetime and current headache (group 1); and *de novo* headache, i.e. individuals in whom headache developed only after cocaine use began (group 2). The correlation between headache and cocaine is controversial and still understimated.

Written informed consent to publish was obtained from the patient(s).

## Conflict of interest

None.
